# Validation and Cross-Cultural Adaptation of the Spanish Version of the Pain Sensitivity Questionnaire (PSQ-S)

**DOI:** 10.3390/jcm11010151

**Published:** 2021-12-28

**Authors:** María del Rocío Ibancos-Losada, María Catalina Osuna-Pérez, Irene Cortés-Pérez, Desirée Montoro-Cárdenas, Ángeles Díaz-Fernández

**Affiliations:** 1 Department of Health Sciences, University of Jaén, Campus Las Lagunillas s/n, 23071 Jaén, Spain; mril0001@red.ujaen.es (M.d.R.I.-L.); icp00011@red.ujaen.es (I.C.-P.); dmc00047@red.ujaen.es (D.M.-C.); andiaz@ujaen.es (Á.D.-F.); 2 Granada Northeast Health District, Andalusian Health Service, Street San Miguel 2, 18500 Guadix, Spain

**Keywords:** pain perception, pain sensitivity, experimental pain testing, questionnaire, chronic pain

## Abstract

Experimental pain testing requires specific equipment and may be uncomfortable for patients. The Pain Sensitivity Questionnaire (PSQ) was developed to assess pain sensitivity, based on the pain intensity ratings (range: 0–10) of painful situations that occur in daily life. The main objective of this study was to carry out a cross-cultural adaptation and validation of the Spanish version of the PSQ (PSQ-S). A total of 354 subjects (296 healthy and 58 chronic pain patients) filled in the PSQ-S. A subgroup of 116 subjects performed experimental pain testing, including two modalities (cold and pressure), with different measures: pain intensity rating, pressure pain threshold, and tolerance. The validation results showed two factors: PSQ-S-moderate and PSQ-S-minor and, for the total scale and the two factors, an excellent internal consistency (Cronbach’s alpha coefficient > 0.9) and a substantial reliability (Intraclass Correlation Coefficient > 0.8). We obtained strong correlations with all the experimental pain rating parameters, catastrophizing, and depression variables, as well as moderate correlations with anxiety, central sensibilization, and impact on the quality of life. Chronic pain patients received elevated PSQ-S scores compared to healthy controls, and three cut-off values (PSQ-S-total = 7.00, PSQ-S-moderate = 7.57, and PSQ-S-minor = 6.29) based on ROC curve analyses were shown to be able to discriminate between healthy adults and adults with chronic pain. Therefore, PSQ-S may be a simple alternative to experimental pain procedures for clinical and experimental pain research.

## 1. Introduction

Chronic pain can be described as “an unpleasant sensory and emotional experience associated with, or resembling that associated with, actual or potential tissue damage that occurs as ongoing pain lasting longer than three months” [1,2,3]. Presently, chronic pain is a major problem in the adult population [4], affecting 11–40% of the global population [2] and causing suffering and disability [5].

For these reasons, the assessment of pain is crucial, as it can provide information concerning the severity of the situation and the pathophysiological mechanism that may be involved [6]. However, pain is a subjective and multidimensional experience. It is difficult to measure it accurately, and this is even more the case for chronic pain due to the complexity of its symptoms, its duration, the difficulty of its treatment, and the pain pathways that may be affected [7]. Pain assessment can address the different domains of pain (location, temporal characteristics, or the sensory and affective qualities of pain), and there are different approaches for assessing pain mechanisms (e.g., biopsies, microneurography, genotyping, pharmacological phenotyping, or quantitative sensory testing) [6].

Specifically, assessing sensitivity to pain (understanding pain sensitivity through the proneness to react to standardized experimental or pathological pain stimuli) [8] is a topic of growing interest with significant clinical and scientific implications. For example, increased pain sensitivity could predict the presence of postoperative pain [9,10,11,12] and the development of chronic pain (e.g., migraines or dysmenorrhea) [13,14,15], and could influence the success of treatment in patients with chronic pain [16,17,18]. Furthermore, it has been shown that this type of patient exhibits a localized and generalized increase in experimental pain perception [19,20,21]. This finding has promoted an understanding of mechanisms underlying chronic pain, suggesting central nervous system mechanisms such as the dysfunction of endogenous pain inhibitory systems [9,22].

However, it should be noted that different factors could influence pain sensitivity: race [23], ethnicity [24], sex [25], age [26], biology (presence of inflammatory molecules) [27], stressors [28], personality [29], sleep [30], profession [31], etc. Comprehending these factors could be helpful in the early identification of the risk of pain and its timely management [32,33]. In addition, gaining a better understanding of how these factors influence pain sensitivity could offer opportunities to control and reduce the impact of pain [28].

One of the most commonly used methods for assessing pain sensitivity is experimental pain testing. However, this method depends on multiple factors, such as the modality of the stimulus (heat, cold, pressure, electrical, ischemic, etc.), its parameters, or its location in the body. Furthermore, implementing a complete program of experimental pain tests could take a great deal of time and require high human and economic costs [34,35,36,37].

In addition to this methodology, it is widely accepted that a self-report is currently the best way to measure pain in adults, through questionnaires in which patients can also reflect on their own experience of pain. Thus, an alternative (or complementary) reliable, valid, time-saving, and cost-saving approach may be assessing pain sensitivity by self-assessment through the Pain Sensitivity Questionnaire (PSQ-S), which was initially developed by Ruth Ruschewayth in 2009 [38].

The PSQ is a self-assessment measure of pain perception based on imaginary painful situations in daily life. It consists of 17 items representing or alluding to common pain situations; each item is scored on a scale from 0 (not painful) to 10 (worst pain imaginable). The PSQ can be summed in total (PSQ-total), in a PSQ moderate subscale or factor (the sum of items 1, 2, 4, 8, 15, 16, and 17, which represent situations of moderate pain), and in PSQ minor (the sum of items 3, 6, 7, 10, 11, 12, and 14, which represent situations of mild pain). The other three items (5, 9, and 13) are not taken into consideration because they represent non-painful situations [38]. Despite the advantages offered by this questionnaire and having been validated in several languages such as English [39], French [40], Mandarin Chinese [41], Polish [42], Korean [43], Iranian [44], Norwegian [45], and Dutch [46], it has not yet been cross-culturally adapted and validated in the Spanish language. Consequently, the principal objective of the study was to carry out a cross-cultural adaptation and validation of the Spanish version of the PSQ (PSQ-S) and analyze its psychometric properties.

## 2. Materials and Methods

### 2.1. Cross-Cultural Adaptation

For the cross-cultural adaptation [47] of the original version of PSQ to the Spanish version, two bilingual experts independently translated the 17 items of this questionnaire into Spanish. By combining these two translations, a first version of the instrument was obtained and subsequently evaluated by three experts from a grammatical and semantic point of view. Based on their comments, they reached a consensus on the PSQ-S. Finally, two other translators, different from the previous ones, transcribed this Spanish version into English to check the consistency between both versions of this measurement instrument.

Afterwards, a total of 10 experts in the field of pain (physiotherapists with more than 10 years of experience and with specific training in chronic pain management) carried out the validation process (following Delphi’s method) [48]. An information collection template was designed for them, which included a report detailing the study’s objective, instructions for participation, and the 14 evaluable items of the questionnaire. The experts could score each item in a range from 1 to 4 according to their degree of relevance (1 ‘not relevant’ and 4 ‘very relevant’). In addition, we asked them to give their opinion about the understanding of the item’s statement and the possibility of including new items. Finally, using the results of the entire group, the Content Validity Index was calculated for each item, with a score above 0.7 considered to be relevant according to the model proposed by Mary R. Lynn [49]. 

The investigation team piloted this questionnaire version in a sample of 15 healthy individuals and patients with Fibromyalgia Syndrome (FMS) to calculate the time taken to complete the questionnaires, detect possible limitations inherent to the questions themselves, and check whether adjustments were necessary. The participants were also asked the following questions: (1) Were any questions unclear to you? (2) Would you modify any questions? (3) Concerning the time it has taken you to answer, would you classify the questionnaire as excessively long, long, standard, or short?

**Figure 1:**
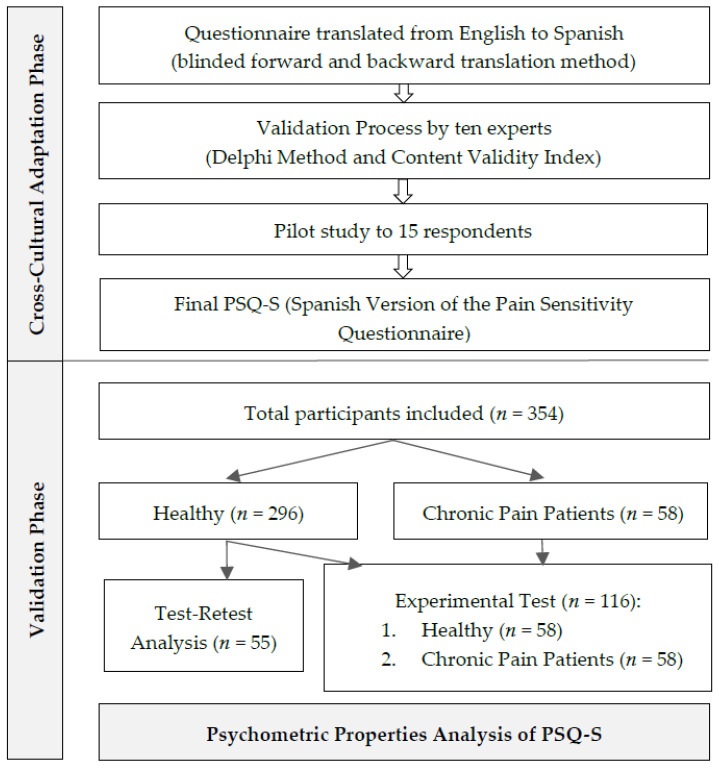
Flowchart of the study.

Figure 1 shows the flowchart of the study.

### 2.2. Participants

We used the criterion to recruit a minimum of 10 subjects per item to calculate the sample size in order to validate the questionnaire. Therefore, a minimum of 140 participants was needed [50]. Recruitment was carried out through advertisements on several social networks and in collaboration with the Fibromyalgia Association of Jaén (AFIXA).

A subsample of the participants was extracted to carry out the experimental tests and for the test–retest analysis. We used a computer random number generator to select them.

The study was conducted with both healthy participants and patients with FMS who met the following inclusion criteria: (a) group of healthy persons: who are over 18 years and have knowledge and understanding of Spanish, absence of daily analgesic medication or drug or alcohol consumption 24 h before the study, and absence of any pain at that time; (b) group of patients with FMS: who must meet the diagnostic criteria for fibromyalgia described by the American College of Rheumatology (ACR) of 2016, are over 18 years of age, and have knowledge and understanding of Spanish.

Exclusion criteria were: pregnancy; cardiac, psychiatric, or neurological disease; fever or infectious diseases; or abnormalities in sensory or mental perception (for example, cerebrovascular accident, hemiparesis, or epilepsy).

The Ethics Committee of the University of Jaén approved the research (DIC.20/6.TES). The study was conducted following the Declaration of Helsinki, good clinical practices, and all applicable laws and regulations. Additionally, all the participants provided written informed consent to participate in the study.

### 2.3. Measurements 

Apart from filling in the PSQ-S, we interviewed all participants to collect demographic data such as their age, sex, education level (Primary, Secondary, or University), and exercise level (exercise hours/week). Additionally, we obtained information about participants’ anxiety, depression symptoms, catastrophizing, central sensitization, and fibromyalgia impact (these last two variables were only measured in the population with chronic pain). Data gathering was completed at the University of Jaén and lasted approximately 20–30 min. The study was performed during December 2020 and February 2021.

Anxiety and depression symptoms were measured using the validated Spanish version of the ‘Hospital Anxiety and Depression Scale’ (HADS) [51]. This scale consists of 14 items—7 for the anxiety subscale and 7 for the depression subscale—with either zero- or three-point Likert-type scales. The test provides two total scores: one for anxiety and the other for depression. Scores range from 0 to 21 in each subscale. Higher scores indicate more anxiety and depressive symptoms. Cronbach’s alpha was 0.81 for the anxiety subscale and 0.87 for the depression subscale.

Catastrophizing was measured with the validated Spanish version of the ‘Pain Catastrophizing Scale’ (PCS) [52]. This is a self-administered 13-item questionnaire in which the subjects indicate the degree to which they experience each of the thoughts or feelings using a five-point Likert scale that ranges from 0 (never) to 4 (always). From the scale, a total score is obtained that reflects the level of catastrophizing in the face of the subject’s pain; the total score range is 13 to 62 points, with low scores indicating a low level of catastrophizing and high values indicating a high level of catastrophizing in the subject. This scale comprises three dimensions: (a) rumination, (b) magnification, and (c) despair. In this study, the complete questionnaire was taken into consideration.

Central sensitization: its presence was measured with the validated Spanish version of the ‘Central Sensitization Inventory’ (CSI) [53]. This is a self-report outcome measure designed to identify patients with symptoms related to central sensitization or central sensitivity syndromes. The CSI includes 25 items, and the score ranges from 0 to 100 points; higher scores indicate more severe symptoms. A score of 40 points has been proposed as the cut-off to determine the presence of central sensitization. The Spanish Version of the CSI demonstrated a high internal consistency (α = 0.872) and test–retest reliability (r = 0.91).

Fibromyalgia impact: to measure physical and psychological symptoms and their interference in daily living tasks in fibromyalgia patients, the validated Spanish version of the ‘Fibromyalgia Impact Questionnaire’ was used (FIQ) [54]. This is a 10-item self-report questionnaire that measures physical disability and the level of specific symptoms such as pain, rigidity, fatigue, depression and anxiety, disability, and general well-being during the last week. Each symptom is rated on a scale from 0 (absence of symptoms) to 10 (very severe). The FIQ score varies from 0 to 100, where higher values indicate a more significant negative impact of the disease. The intraclass correlation coefficient for the Spanish-FIQ was 0.81, and the S-FIQ scores obtained a good correlation with the SF-36 scores.

A subsample of randomly chosen participants completed the PSQ-S questionnaire three weeks later. Another subsample composed of healthy and FMS participants also carried out the two experimental tests in the laboratories of the University of Jaén, for a total duration of approximately 20 min. Participation in the experimental tests yielded new study variables, as described below:

Cold Pressor Test [40,45,55]: the tolerance (‘Tolerance CPT’ variable) and intensity of pain from cold water (‘Intensity Pain CPT’ variable) were explored, by immersing the non-dominant foot in a water bath at 3 °C for a maximum time of two minutes. During the stimulus, the subjects rated the intensity of pain from 0 to 10 every 30 s, with a score of 0 being no pain and 10 being the worst pain imaginable (the mean of the different scores equaled the pain intensity variable in the cold water test). The time taken to remove the foot from cold water was measured and served as a measure of tolerance to cold water. Pain ratings missing after abstinence for up to two minutes were replaced by a score of ten, signifying the worst pain imaginable. Participants also had to rate (0–10) how unpleasant the stimulus had been, with 0 being not unpleasant and 10 being the most unpleasant you can imagine (‘unpleasant CPT’ variable) [56].

Pain by algometry: pressure pain threshold (‘PPT’ variable) was measured (kg/cm²). For this purpose, we used a digital algometer with a 1.0 cm² probe (Wagner Instruments, Force TENTM Digital Force Gage FDX 50, Greenwich, CT, USA). The pressure was applied to three different points on the neck (two on the trapezius muscle and one on the scalene muscle) following the same sequence [57]. The investigator applied the pressure and the participants indicated when the sensation of pressure changed to pain, at which point the investigator immediately stopped the stimulus and noted the pressure supported. The mean pressure score served to determine the ‘PPT’ variable. Additionally, participants had to rate how unpleasant the stimulus had been (0–10), with 0 being not unpleasant and 10 being the most unpleasant sensation you could imagine (‘unpleasant PPT’ variable) [56].

### 2.4. Statistic Analysis

Data were analyzed using the SPSS 21.00 statistical package (SPSS Inc., Chicago, IL, USA). The level of significance was set at 0.05 for all tests and the confidence interval at 95% [58].

Data were described using mean and standard deviation for continuous variables as well as frequencies and percentages for categorical variables. The Kolmogorov–Smirnov test was used to determine the normality of the continuous variables.

The construct validity was analyzed by exploratory factorial analysis (factorial validity) using principal component analysis (PCA) with Varimax rotation. In addition, Bartlett’s sphericity test and the Kaiser–Meyer–Olkin test (KMO) were administered to test the suitability of the sample for performing a factorial analysis [59].

Internal consistency was measured using Cronbach’s alpha coefficient; it was considered poor if it was less than 0.70, good if it was between 0.70 and 0.90, and excellent if it was greater than 0.90. 

The test–retest reliability of the total test score was measured using the Shrout and Fleiss type 2.1 intraclass correlation coefficient (ICC). Reliability was considered poor when the ICC was <0.40, moderate when the ICC was between 0.40 and 0.75, substantial when the ICC was between 0.75 and 0.90, and excellent when the ICC was >0.90 [60].

We used Pearson’s correlation coefficient r to analyze the convergent validity of PSQ-S scores with the HADS and PCS punctuations, the concurrent validity of PSQ-S scores with the experimental variables (‘Intensity Pain CPT’, ‘Tolerance CPT’, and ‘PPT’), and the correlation between PSQ-S scores and the ‘unpleasant CPT’, ‘unpleasant PPT’, FIQ, and CSI variables. This correlation coefficient was considered ‘strong’ if it was >0.50 and ‘moderate’ between 0.30 and 0.50.

To test the differences regarding pain sensitivity between FMS patients and healthy individuals, a one-way analysis of variance (ANOVA) was used, where the mean scores of the two populations (FMS and healthy) were compared for each factor of PSQ-S.

Finally, the discriminatory ability of the PSQ-S to distinguish between healthy controls and chronic pain patients (subjects with and without FMS) was determined using the receiver operating characteristic (ROC) methodology. In the ROC curve, the true positive rate (Sensitivity) was plotted as a function of the false positive rate (100-Specificity) for different cut-off points. Each point on the ROC plot represented a sensitivity/specificity pair corresponding to a particular decision threshold. A test with perfect discrimination would have a ROC curve passing through the top left-hand corner (100% sensitivity and 100% specificity). The area under the curve (AUC) was also calculated as a measure of the ability of the score (total score and each factor score) to discriminate between the two groups (healthy and chronic pain subjects) [61]. The AUC value was considered statistically significant when the 95% CI did not include a 0.5 value. Values between 0.5 and 0.7 indicated a low accuracy, values between 0.7 and 0.9 indicated a good accuracy, and values greater than 0.9 indicated a high accuracy [62]. We used the method developed by Hanley and McNeil to calculate the standard error of the AUC, while the exact binomial test was used to calculate the CI for the AUC [63]. Additionally, examining the intersections of the sensitivity and 1-specificity plots nearest to the upper left corner of the graph, the optimal cut-off values for maximal average sensitivity and the specificity for detecting change were identified for the three subscales of the PSQ-S (PSQ-S-total, PSQ-S-moderate, and PSQ-S-minor).

## 3. Results

### 3.1. Cross-Cultural Adaptation of the PSQ-S

After the translation and backward translation process, the committee of experts (who participated in the Delphi method) concluded that no item should be deleted or added; they only advised that the expression of items 7, 10, and 11 should be modified. Item 7, ‘Imagine you graze your knee falling off the bicycle’, was changed to ‘Imagine that you scrape your knee when you hit the ground’, to describe a more general situation, as some subjects may have never ridden a bicycle. Item 10, ‘Imagine you have a minor cut on a finger and inadvertently get lemon juice in the wound’, was changed to ‘Imagine you have a minor cut on a finger and inadvertently get salt in the wound’, as this seemed to be a more common situation. Item 11, ‘Imagine you prick your fingertip on the thorn of a rose’, was changed to ‘Imagine you grasp a rose and prick yourself with a thorn from the stem that you had not seen’, to emphasize the situation more. 

After these minor changes, it was not considered that any item should be added or removed. The Content Validity Index was found to be higher than 0.7 for all items. 

The pilot study was carried out without problems, showing that all items were understood, no modification of the questionnaire was proposed, and the completion time was short (participants took between 5–10 min). Finally, the PSQ-S was obtained for the validation phase. 

### 3.2. Participants 

A total of 354 people participated, with 296 people belonging to the group of healthy participants and 58 belonging to the group of participants with FMS. A subsample of 55 healthy participants performed the retest assessment, and a subsample of 116 participants (both healthy and chronic pain patients) was extracted to carry out the experimental tests. Table 1 shows the sociodemographic and basic data for the sample. 

The majority of healthy participants were women (62.5%), with a mean age of 34.2 ± 9.74 years; the majority of this group has studied at university (88.2%), presented a BMI of 24.26 ± 4.19, and performed an average of 5.44 ± 6.23 h of exercise a week. This group obtained low scores on the depression (3.65 ± 2.88), anxiety (6.62 ± 3.31), and catastrophism (12.48 ± 9.06) scales.

In the group of patients with chronic pain (FMS), the majority were also women (86.2%) but with a significantly higher mean age (52.97 ± 9.37 years), and only 17.2% had completed university studies. They presented a BMI of 27.62 ± 5.55 and performed fewer hours of exercise per week (3.69 ± 2.6). This group of patients with chronic pain showed high scores in all the administered questionnaires for anxiety (13.78 ± 3.39), depression (10.34 ± 4.41), catastrophism (31.14 ± 11.10), and central sensitization (71.39 ± 11.38). They also presented a high impact of the disease on their quality of life (75.33 ± 14.91). 

### 3.3. Construct Validity

The factorial analysis by principal components showed a good KMO measure (KMO = 0.958, Chi-square = 3904.338, *p* < 0.001), indicating that the sample of patients was adequate for the analysis. The factorial analysis of the data extracted two factors that explained 69% of the variance. The Varimax rotation grouped the items into the same two recognizable factors of the original version: PSQ-moderate (made up of seven items that refer to situations of intense–moderate pain: items 1, 2, 4, 8, 15, 16, and 17) and PSQ-minor (made up of seven items that refer to situations of mild pain: items 3, 6, 7, 10, 11, 12, and 14). Every item was loaded >0.6 on one of the two factors (Table 2).

### 3.4. Internal Consistency

The Cronbach’s Alpha scores for PSQ-S-total, PSQ-S-moderate, and PSQ-S-minor were 0.95, 0.91, and 0.92, respectively, indicating an excellent internal consistency. In addition, the analysis of the items (Table 2) showed that all items seemed to contribute adequately to the consistency of the questionnaire. 

### 3.5. Test–Retest Reliability

Healthy participants who underwent the experimental tests were invited to participate in a retest assessment three weeks later. The test–retest reliability was assessed in 55 participants. The Intraclass Correlation Coefficient was 0.84 for PSQ-S-total, 0.82 for PSQ-S-moderate, and 0.84 for PSQ-S-minor. The intraclass correlation was also significant for all items (Table 2).

### 3.6. Convergent Validity

The convergent validity of the PSQ-S-total was strong with catastrophizing; moderate with depression, anxiety, and central sensitization; and poor with the impact on quality of life. 

The PSQ-S-moderate factor had a strong correlation with catastrophizing, a moderate correlation with depression and anxiety, and a poor correlation with central sensitization, but no correlation was found with the impact on quality of life.

The PSQ-S-minor had a strong correlation with depression and catastrophizing and a moderate correlation with anxiety, central sensitization, and the impact on quality of life (Table 3).

### 3.7. Concurrent Validity

We assessed the concurrent validity by comparing the results of the experimental tests with the PSQ-S scores. For this, we drew a sample of 116 individuals (58 healthy people and 58 people with FMS).

We obtained a strong correlation with the intensity of pain from cold water, the tolerance to the Cold Pressor Test, and the Pressure Pain Threshold in the total score and each factor (PSQ-S-moderate and PSQ-S-minor). These results are shown in Table 4. 

A moderate association was found between the sensation of the unpleasantness of the stimuli (cold water and pressure) and the scores for PSQ-S-total, PSQ-S-moderate, and PSQ-S-minor (Table 4).

### 3.8. Comparison of PSQ-S Scores and Experimental Variables between Chronic Pain Patients and Healthy Controls

The ANOVA (Table 5) showed statistically significant differences between the groups (healthy patients and those with chronic pain) for the total PSQ-S and in each factor. Additionally, we found statistically significant differences in the pain intensity in CPT, tolerance to cold water (seconds), unpleasant sensation from cold water (check from 0–10), PPT, and the unpleasant feeling of pressure pain (update 0–10). 

The healthy population showed greater pain tolerance or lower sensitivity in the three PSQ-S scores than the FMS population.

A sample of 116 people (58 in each group) was subtracted to carry out the experimental tests. Again, the healthy population showed a greater tolerance to pain, enduring longer in CPT, qualifying this test with a lower score, and obtaining a higher PPT than those in the FMS group.

We also found differences in how unpleasant the stimuli applied (cold water and pressure) had been; the population with FMS reported higher scores regarding how unpleasant the sensation had been.

### 3.9. ROC Curve Analysis

The ROC curve analysis showed an AUC value of 0.926 (CI = 0.894 to 0.951, *p* < 0.001) when discriminating between FMS patients (in terms of subjects with chronic pain) and healthy individuals. It also showed that cut-off values of more than 7 points in the PSQ-S-total could discriminate between healthy adults and adults with chronic pain. In this respect, the PSQ-S demonstrated high levels of sensitivity (79.31%) and specificity (96.28%) for identifying chronic pain patients, with high positive and negative predictive values (+PV = 80.7; −PV = 96.0). 

Analyzing the PSQ-S-moderate factor, the ROC curve analysis showed an AUC of 0.884 (CI = 0.846 to 0.0.915, *p* < 0.001) and cut-off values of more than 7.57 points in this factor with a sensitivity of 77.59% and a specificity of 90.88%. The high positive and negative predictive values were 62.5 and 95.4, respectively.

Analyzing the PSQ-S-minor factor, the ROC curves analysis showed an AUC of 0.941 (CI = 0.911 to 0.0.963, *p* < 0.001) and cut-off values of more than 6.29 points in this factor, with a sensitivity of 77.59% and a specificity of 96.28%. The high positive and negative predictive values were 80.4 and 95.6, respectively.

**Figure 2:**
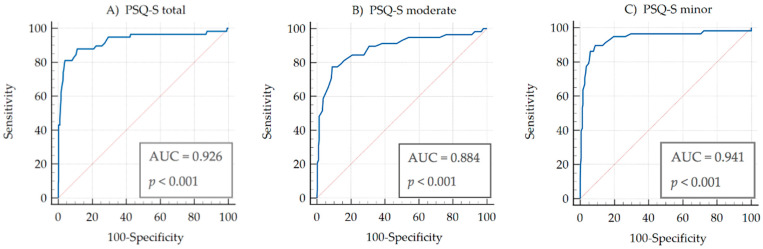
The capacity of PSQ-S to discriminate between the sensitivity of chronic pain patients and healthy controls: cut-off points and their predictive values.

Figure 2 and Table 6 show all the ROC curve analysis data of the PSQ-S.

## 4. Discussion

This investigation translated the original version of the PSQ into the Spanish language, performed a transcultural adaptation, and validated it in a Spanish population (Appendix A).

Compared with other PSQ validations, the structure of the items remained as in most of them, although some cultural adaptations in several items had to be made (such as in the Chinese version of the PSQ [41]). In addition, the participants to perform our validation consisted of healthy participants, as in all the other adaptations of the questionnaire (Norwegian, German, French, Chinese, Iranian, or Dutch versions [38,40,41,44,45,46]). Nevertheless, our study also included individuals with FMS, a population whose PSQ studies have not yet been conducted.

Our study observed demographic differences between both groups: the population with FMS was older, and was made up of a high percentage of women who had generally not completed university studies and were overweight, as found in other studies carried out in FMS populations [64]. Additionally, we observed differences in the anxiety, depression, and catastrophizing scores, which were higher in the population with FMS (characteristics described in FMS [65]). 

In the validation phase, the factor analysis extracted two factors that perfectly matched the two subscales (PSQ-moderate and PSQ-minor) established in the original study. In addition, every item had a high and clear load for its factor that was higher than in the original version [38].

An excellent internal consistency was obtained with the Cronbach’s alpha for PSQ-S-total, PSQ-S-moderate, and PSQ-S-minor. The results obtained were similar to those found for the original, Norwegian, French, Chinese, Korean, Iranian, Dutch, and Polish versions [40,41,42,43,44,45,46].

The analysis indicated a substantial test–retest reliability. The ICC values were higher than those obtained in the French, Chinese, and Korean versions [40,41,43]. However, they were very similar to the original and the Iranian versions [38,44]. In the same way, our ICC results were slightly lower than those found in the Polish version, although it should be noted in that version the mean retest time was nine days, while the retest time in our study was three weeks [42] (Appendix B: Summary of the results of PSQ validation studies in other languages).

Strong and positive correlations were found between PSQ-S-total, PSQ-S-moderate, and PSQ-S-minor with PCS. In the German, French, English, and Korean versions, moderate correlations were found [38,39,40,43], while weak correlations were found in the Chinese version [41]. Only the Iranian version obtained an excellent correlation [44].

We found moderate and positive correlations among PSQ-S-total, PSQ-S-moderate, and PSQ-S-minor with the anxiety variable. In addition, the German version had a moderate correlation with PSQ-minor [38], the French version found a weak correlation [40], and in the English and Chinese versions no correlations were found [39,41].

Moderate and positive correlations were found between PSQ-S-total and PSQ-S-moderate with the depression variable, but a strong correlation was found for PSQ-S-minor. The German version obtained a moderate relation with PSQ-minor [38] and the French version found a weak correlation [40]. No correlations were found in the English and Chinese versions [39,41].

Moderate and positive correlations were found between the PSQ-S-total and PSQ-S-minor scores with CSI (r = 0.33 and 0.36, respectively), but a poor correlation was found with PSQ-S-moderate (r = 0.29). A moderate and positive correlation was found between the FIQ and PSQ-S-minor, a poor correlation was found with PSQ-S-total, and no correlation was found with PSQ-S-moderate. Other PSQ validations have not analyzed these correlations, but there is evidence of the relationship between pain sensitivity, central sensitization, and quality of life [66,67].

Regarding the evaluation of concurrent validity, we found strong and negative correlations between PSQ-S-total, PSQ-S-moderate, and PSQ-S-minor with ‘PPT’ (r = −0.59, −0.50, and −0.60, respectively) and ‘tolerance CPT’ (r = −0.56, −0.52, and −0.57, respectively). Additionally, we obtained strong and positive correlations with ‘pain intensity CPT’ (r = 0.65, 0.57, and 0.60, respectively). The original study did not find correlations with PPT, but moderate correlations were obtained with pain intensity CPT (r = 0.42, 0.35, and 0.45, respectively) [38]. The French version obtained weak correlations during the first 30 s in CPT [40]. The Norwegian version also analyzed correlations with ‘tolerance CPT’ and with ‘CPT pain intensity’, but the strength of the association was only moderate in both variables [45].

Moderate and positive correlations were found among the three scores of PSQ-S and the ‘unpleasant CPT’ (r = 0.40, 0.39, and 0.39, respectively) and the ‘unpleasant PPT’ variables (r = 0.41, 0. 41, and 0.40, respectively). Although no previous studies have analyzed this relationship, some studies have examined the influence of this variable on pain modulation processes and suggested that the degree of affinity to a specific stimulus could interact in these processes [56,57]. 

It is possible that other studies did not find a strong correlation between CPT and the other variables because these populations are accustomed to cold, and, therefore, they could have a greater tolerance to cold and would be adapted to this stimulus. For this reason, the measurement of the ‘unpleasant variable’ is recommendable because the affinity for the stimulus could explain many of the results (high affinity for the stimulus would imply a greater tolerance to it and vice versa) [56,57].

The correlations show that the Spanish questionnaire version of the PSQ has a good concurrent and convergent validity and that pain sensitivity is associated with variables of the cognitive–emotional dimension (anxiety, depression, catastrophism, and affinity to painful stimuli).

In our investigation, the total, moderate, and minor PSQ-S scores were 4.52 ± 1.47, 5.59 ± 1.59, and 3.46 ± 1.56, respectively, for the healthy population. If we compare the healthy populations of the different versions, the Spanish version showed similar pain scores with minimal differences to those of the German, Dutch, French, Chinese, and Norwegian versions [38,40,41,45,46]. These differences may have been caused by cultural differences between the subjects [24]. The PSQ-S scores for the FMS population were 7.67 ± 1.58, 8.08 ± 1.53, and 7.26 ± 1.71, respectively, exposing a significant difference between both populations, where the population with FMS shows high scores, and, therefore, shows a greater sensitivity to pain or less tolerance to it compared to healthy individuals. In addition, in the healthy population there is a clear difference in the mean scores of both factors, showing that this group discriminates well between situations of intense pain and situations of mild pain. The two factors of the PSQ-S, therefore, are well-reflected. However, there is a slight difference between the mean scores for both factors in patients with fibromyalgia. In general, all the situations raised were very painful for these patients, and they did not have the ability to discriminate between mild and moderate pain situations. The central sensitization characteristic of FMS could explain this hypersensitivity to pain and lack of nuance in its perception [66].

The sensation of the unpleasantness of the stimuli (cold water and pressure) was also measured, with the FMS population experiencing these stimuli as more unpleasant than the healthy group. These variables have an emotional and individual component (a preference based on previous experience or their own beliefs); for this reason, they are considered to belong to the cognitive–emotional sphere and could intervene negatively in the pain modulation processes [68]. On the other hand, these variables may be influenced by the sensitivity to pain itself, which we already know is higher in this population [66], therefore, making the ‘unpleasant’ scores higher. Future research should analyze the influence of these variables on the process of sensitivity to pain and its greater or lesser impact.

Finally, the result of the ROC curves analysis establishes a cut-off value of 7.00 points in PSQ-S-total, 7.57 in PSQ-S-moderate, and 6.29 in PSQ-S-minor. These data allow us to discriminate between pain sensitivity in patients with chronic pain and healthy individuals, showing good values regarding the sensitivity of the questionnaire (>75%) and its specificity (>90%). Thus, these results support the notion that PSQ-S can be used as a complementary tool for assessing and detecting the alarm signal in individuals for whom there is suspicion that a chronic pain process could be occurring. This is the first time in PSQ validation studies that the ROC methodology has been used.

The present study had some limitations. Not all painful modality stimuli were used. Other forms of Quantitative Sensory Testing (e.g., heat, ischemic, or electrical) should be evaluated in future research. The item that asks participants about snow (item 12) may not apply in Spanish areas where the climate is warm. However, this item has shown a good correlation with the total score. Other chronic pain populations, in addition to fibromyalgia patients, should be evaluated.

## 5. Conclusions

The PSQ-S has excellent psychometric properties, reliability, and stability as well as an identical structure to the original version. Therefore, this questionnaire could be considered a simple, useful, and effective alternative to experimental tests that study pain sensitivity, saving time and resources, as it only requires 5–10 min to complete. It could also constitute a complementary tool for assessing and detecting chronic pain processes in clinical practice.

## Figures and Tables

**Table 1:** Descriptive characteristics for the total cohort and the two populations independently.

Variables	Total Cohort (*n*= 354)	Healthy (*n* = 296)	Chronic Pain Patients (*n* = 58)	*p*-Value
Sex				0.001
Male	119 (33.6)	111 (37.5)	8 (13.8)	
Female	235 (66.4)	185 (62.5)	50 (86.2)	
Education level				0.001
Primary	15 (4.2)	0 (0)	15 (25.9)	
Secondary	68 (19.2)	35 (11.9)	33 (57)	
University	271 (76)	261 (88.2)	10 (17.2)	
Age (years)	37.27 ± 11.91	34.2 ± 9.74	52.97 ± 9.37	0.001
BMI (kg/m2)	24.81 ± 4.61	24.26 ± 4.19	27.62 ± 5.55	0.001
Exercise (hours)	5.16 ± 5.83	5.44 ± 6.23	3.69 ± 2.6	0.001
HADS total (0–42)	12.54 ± 7.68	10.27 ± 5.42	24.12 ± 7.11	0.001
HADS anxiety (0–21)	7.79 ± 4.25	6.62 ± 3.31	13.78 ± 3.39	0.001
HADS depression (0–21)	4.75 ± 4.03	3.65 ± 2.88	10.34 ± 4.41	0.001
PCS (0–52)	15.54 ± 11.67	12.48 ± 9.06	31.14 ± 11.10	0.001
FIQ (0–100)	-	Not applicable	75.33 ± 14.91	-
CSI (0–100)	-	Not applicable	71.39 ± 11.38	-

Data are given as means and standard deviations for continuous variables and frequencies (percentages) for categorical variables. A one-way ANOVA was used to analyze the distribution of the quantitative variables and the chi-square test was used for categorical variables. BMI: Body Mass Index; HADS: Hospital Anxiety and Depression Scale; PCS: Pain Catastrophizing Scale; FIQ: Fibromyalgia Impact Questionnaire; CSI: Central Sensitization inventory.

**Table 2:** Item analysis.

Items	Loaded PSQ-S- Moderate (*n* = 354)	Loaded PSQ-S- Minor (*n* = 354)	Corrected Correlation Item-Total (*n* = 354)	Cronbach Alpha If Item Is Deleted (*n* = 354)	Test–Retest Correlation ICC (*n* = 55)
Item 1	**0.783**	0.267	0.697	0.952	0.71
Item 2	**0.693**	0.358	0.699	0.952	0.60
Item 3	0.340	**0.745**	0.729	0.951	0.72
Item 4	**0.724**	0.377	0.736	0.951	0.76
Item 6	0.261	**0.847**	0.748	0.951	0.81
Item 7	0.384	**0.786**	0.796	0.950	0.74
Item 8	**0.627**	0.538	0.786	0.950	0.75
Item 10	0.455	**0.724**	0.802	0.950	0.85
Item 11	0.464	**0.707**	0.796	0.950	0.81
Item 12	0.496	**0.609**	0.745	0.951	0.72
Item 14	0.455	**0.693**	0.781	0.950	0.78
Item 15	**0.708**	0.386	0.734	0.951	0.78
Item 16	**0.750**	0.397	0.775	0.950	0.85
Item 17	**0.755**	0.393	0.774	0.950	0.84

Loaded items for each factor obtained by principal component analysis with Varimax rotation (bold indicates loads > 0.6 in one of the two factors). Correlation of the item with the total score. Cronbach’s Alpha is shown if the item is deleted. ICC: Intraclass Correlation Coefficient.

**Table 3:** Convergent validity measured by Pearson correlation.

	PSQ-S Total	PSQ-S Moderate	PSQ-S Minor
**HADS depression** (*n* = 354)	0.49 **	0.41 **	0.52 **
**HADS anxiety** (*n* = 354)	0.45 **	0.37 **	0.49 **
**PCS** (*n* = 354)	0.58 **	0.50 **	0.60 **
**FIQ** (*n*= 58)	0.26 *	0.19	0.31 **
**CSI** (*n* = 58)	0.33 **	0.29 *	0.36 **

* *p* < 0.05; ** *p* < 0.01. HADS: Hospital Anxiety and Depression Scale; PCS: Pain Catastrophizing Scale; FIQ: Fibromyalgia Impact Questionnaire; CSI: Central Sensitization Inventory.

**Table 4:** Correlations between PSQ-S scores and single experimental pain rating parameters (*n* = 116).

	PSQ-S-Total	PSQ-S-Moderate	PSQ-S-Minor
Pain intensity CPT (0–10)	0.65 **	0.57 **	0.60 **
Tolerance CPT (in seconds)	−0.56 **	−0.52 **	−0.57 **
Unpleasant CPT (0–10)	0.40 **	0.39 **	0.39 **
PPT (kg/cm²)	−0.59 **	−0.50 **	−0.60 **
Unpleasant PPT (0–10)	0.41 **	0.41 **	0.40 **

** *p* = 0.01. Correlations between PSQ-S scores and single experimental pain rating parameters. Values are Pearson’s correlation coefficients. CPT: Cold Pressor Test. PPT: Pressure Pain Threshold.

**Table 5:** Between-group differences in factor components of PSQ-S and the experimental pain sensitivity testing.

	Healthy *n* = 296		Chronic Pain Patients (FMS) *n* = 58		ANOVA
	Mean	SD	Mean	SD	*p*-Value
PSQ-S-total (0–10)	4.52	1.47	7.67	1.58	<0.001
PSQ-S-moderate (0–10)	5.59	1.59	8.08	1.53	<0.001
PSQ-S-minor (0–10)	3.46	1.56	7.26	1.71	<0.001
	**Healthy** ***n* = 58**		**Chronic Pain Patients (FMS) *n* = 58**		***p*-Value**
Pain intensity CPT (0–10)	6.93	2.16	9.4	0.74	<0.001
Tolerance CPT (in seconds)	91.24	37.84	36.45	32.99	<0.001
Unpleasant CPT (0–10)	6.33	2.13	7.67	2.15	<0.001
PPT (kg/cm²)	1.80	0.68	0.91	0.45	<0.001
Unpleasant PPT (0–10)	2.22	1.72	4.33	2.71	<0.001

CPT: Cold Pressor Test. PPT: Pressure Pain Threshold. FMS: Fibromyalgia Syndrome.

**Table 6:** ROC curve analysis data of the PSQ-S.

	Criterion	Sensitivity	95% CI	Specificity	95% CI	+PV	95% CI	−PV	95% CI
**PSQ-S-total**	>7	79.31	66.6–88.8	96.28	93.4–98.1	80.7	69.8–88.3	96.0	93.5–97.5
**PSQ-S-moderate**	>7.57	77.59	64.7–87.5	90.88	87.0–93.9	62.5	53.1–71.0	95.4	92.8–97.1
**PSQ-S-minor**	>6.29	77.59	64.7–87.5	96.28	93.4–98.1	80.4	69.3–88.1	95.6	93.1–97.3

95% CI: 95% confidence interval; +PV: positive predictive value; −PV: negative predictive value.

## Data Availability

Data available under request to corresponding author due to participants’ consent.

## Abbreviations

ACR: American College of Rheumatology; AUC: Area Under the Curve; BMI: Body Mass Index; CPT: Cold Pressor Test; CSI: Central Sensitization inventory; FIQ: Fibromyalgia Impact Questionnaire; FMS: Fibromyalgia Syndrome; HADS: Hospital Anxiety and Depression Scale; ICC: Intraclass Correlation Coefficient; KMO: Kaiser–Meyer–Olkin test; PSQ: Pain Sensitivity Questionnaire; PSQ-S: Spanish Version of the Pain Sensitivity Questionnaire; PCA: Principal Component Analysis; PCS: Pain Catastrophizing Scale; PPT: Pressure Pain Threshold; ROC: Receiver Operating Characteristic.

## Appendix A. Pain Sensitivity Questionnaire (Spanish Version)

A continuación se van a exponer una serie de situaciones dolorosas que hemos podido sufrir u observar a lo largo de nuestra vida. Puntúe del 0 al 10 cómo de dolorosas cree que le resultaría (0 sería nada doloroso y 10 lo más doloroso que podría imaginar). No hay respuestas buenas ni malas, conteste en base a lo que sería su propia percepción de dolor de estas situaciones imaginadas.

1. Imagina que te golpeas la espinilla violentamente con un borde duro, por ejemplo, con el borde de una mesa de cristal._____(0–10)
2. Imagina que te quemas la lengua con una bebida ardiendo. ______(0–10)
3. Imagina que los músculos te duelen levemente como resultado de una actividad física._____(0–10)
4. Imagina que te pillas el dedo con un cajón. ______(0–10)
5. Imagina que te duchas con agua tibia.______(0–10)
6. Imagina que te has quemado un poco los hombros por el sol.______(0–10)
7. Imagina que te raspas la rodilla al caer al suelo.______(0–10)
8. Imagina que accidentalmente te muerdes la lengua o la mejilla mientras comes._____(0–10)
9. Imagina que caminas descalzo por un suelo de baldosas frías._____(0–10)
10. Imagina que tienes una pequeña herida por un corte en un dedo y sin darte cuenta te entra sal._____(0–10)
11. Imagina que agarras una rosa y te pinchas con una espina del tallo que no habías visto._____(0–10)
12. Imagina que metes las manos desnudas (sin guantes) en la nieve durante un par de minutos o que las pones en contacto con la nieve algún tiempo, por ejemplo, mientras haces bolas de nieve._____(0–10)
13. Imagina que le das la mano a alguien que te aprieta lo normal._____(0–10)
14. Imagina que le das la mano a alguien que te aprieta muy fuerte._____(0–10)
15. Imagina que, inadvertidamente, agarras una olla caliente por sus asas que están igualmente calientes._____(0–10)
16. Imagina que llevas sandalias y alguien con botas pesadas te pisa el pie._____(0–10)
17. Imagina que te das un golpe con el codo en el borde de una mesa (“dolor de la suegra”)._____(0–10)

## Appendix B. Summary of the Results of PSQ Validation Studies in Other Languages

	**Original** **German**	**Norwegian**	**French**	**Chinese**	**Korean**	**Iranian**	**Dutch**	**Polish**	**Spanish**
Internal Consistency (Cronbach’s alpha)									
PSQ-total	0.92	0.92	0.93(P) 0.85(H)	0.90	0.93	0.81	0.90	0.96	0.95
PSQ-moderate	0.81	0.90	0.87(P) 0.87(H)	0.86	0.87	0.82	0.86		0.91
PSQ-minor	0.91	0.85	0.87(P) 0.91(H)	0.81	0.88	0.82	0.82		0.92
Reliability test-retest (ICC)									
PSQ-total	0.83		0.62	0.73	0.78	0.84		0.93	0.84
PSQ-moderate	0.86		0.62	0.74	0.79	0.84		0.87	0.82
PSQ-minor	0.79		0.63	0.68	0.75	0.85		0.81	0.84
Correlation with PCS (r)									
PSQ-total	0.45			0.27	0.38	0.81			0.58
PSQ-moderate	0.43			0.23	0.36				0.50
PSQ-minor	0.38			0.27	0.37				0.60
Correlation with Anxiety (r)									
PSQ-total			0.24						0.45
PSQ-moderate			0.23						0.37
PSQ-minor	0.34		0.24						0.49
Correlation with Depression (r)									
PSQ-total			0.26						0.49
PSQ-moderate			0.22						0.41
PSQ-minor	0.39		0.29						0.52
Correlation with pain intensity CPT									
PSQ-total	0.42	0.36	0.24						0.65
PSQ-moderate	0.35	0.36	0.14						0.57
PSQ-minor	0.45	0.30	0.22						0.60
PSQ scores (Healthy)									
PSQ-total	3.6 ± 1.2	4.5 ± 1.5	4.1 ± 1.2	4.7 ± 1.6			4.1 ± 1.3		4.52 ± 1.47
PSQ-moderate	4.7 ± 1.6	5.9 ± 1.8	5.2 ± 1.3	5.5 ± 1.9			5.3 ± 1.5		5.59 ± 1.59
PSQ-minor	2.5 ± 1.1	3.1 ± 1.3	3.0 ± 1.3	3.9 ± 1.6			2.8 ± 1.3		3.46 ± 1.56

P: Patients; H: Healthy participants; ICC: Intraclass Correlation Coefficient; PCS: Pain Catastrophizing Scale; (r): Pearson’s correlation coefficient; CPT: Cold Pressor Test.
